# Effects of angiotensin-converting enzyme inhibitors or angiotensin receptor blockers on all-cause mortality, cardiovascular death, and cardiovascular events among peritoneal dialysis patients: A protocol for systematic review

**DOI:** 10.1097/MD.0000000000019767

**Published:** 2020-04-24

**Authors:** Surapon Nochaiwong, Chidchanok Ruengorn, Pajaree Mongkhon, Kednapa Thavorn, Ratanaporn Awiphan, Kajohnsak Noppakun, Surachet Vongsanim, Wilaiwan Chongruksut, Brian Hutton, Manish M. Sood, Greg A. Knoll

**Affiliations:** a Department of Pharmaceutical Care; b Pharmacoepidemiology and Statistics Research Center (PESRC), Faculty of Pharmacy, Chiang Mai University, Chiang Mai; c Division of Pharmacy Practice, Department of Pharmaceutical Care, School of Pharmaceutical Sciences, University of Phayao, Phayao, Thailand; d Ottawa Hospital Research Institute, Ottawa Hospital; e Institute of Clinical and Evaluative Sciences, ICES uOttawa; f School of Epidemiology and Public Health, Faculty of Medicine, University of Ottawa, Ottawa, Ontario, Canada; g Department of Internal Medicine, Division of Nephrology; h Department of Surgery, Faculty of Medicine, Chiang Mai University, Chiang Mai, Thailand; i Division of Nephrology, Department of Medicine, University of Ottawa, Ottawa, Ontario, Canada.

**Keywords:** cardiovascular, end-stage kidney disease, mortality, peritoneal dialysis, renin-angiotensin system, systematic review

## Abstract

**Background:**

Based on the International Society for peritoneal dialysis (PD) recommendations, blockade of renin-angiotensin systems with an angiotensin-converting enzyme inhibitors (ACEI) and angiotensin receptor blockers (ARB) improves residual kidney function in PD patients. However, the long-term effectiveness of ACEI/ARB use in PD patients has not been fully elucidated. We, therefore, intend to perform a systematic review and meta-analysis to summarize the effects of ACEI/ARB use on long-term mortality, cardiovascular outcomes, and adverse events among PD patients.

**Methods:**

This systematic review will include both randomized controlled trials and non-randomized studies in adult PD patients. We also plan to incorporate data from our cohort study in Thai PD population into this review. We will search PubMed, Medline, EMBASE, Cochrane Library, Web of Science, Scopus, CINAHL, and grey literature from inception to February 29, 2019, with no language restrictions. The process of study screening, selection, data extraction, risk of bias assessment, and grading the strength of evidence will be performed independently by a pair of reviewers. Any discrepancy will be resolved through a team discussion and/or consultation with the third reviewer. The pooled effects estimate and 95% confidence intervals will be estimated using DerSimonian-Laird random-effects models. Heterogeneity will be assessed by the Cochran Q test, *I*^2^ index and tau-squared statistics. The funnel plots along with the Begg and Egger test and trim and fill method will be performed to investigate any evidence of publication bias. Preplanned subgroup analyses and random-effects univariate meta-regressions will be performed to quantify the potential sources of heterogeneity based on studies- and patient-characteristics.

**Results:**

This will be the first systematic review and meta-analysis to summarize the long-term effectiveness of renin-angiotensin system inhibitors in PD populations.

**Conclusion:**

In summary, this systematic review and meta-analysis will summarize the effectiveness of ACEI/ARB on long-term mortality, cardiovascular outcomes, and adverse events among adult PD patients by integrated all available evidences.

**Ethics and dissemination:**

Based on the existing published data, an ethical approval is not required. The findings will be disseminated through scientific meetings and publications in peer-reviewed journals.

PROSPERO registration number: CRD42019129492.

## Abstract 2

Supplemental Digital Content is available in the text

## 1 Introduction

Peritoneal dialysis (PD) is a well-established treatment option of home renal replacement therapy for end-stage kidney disease (ESKD) patients. Approximately, 11% with more than 272,000 individuals with ESKD patients undergo PD as renal replacement therapy in worldwide.^[1,2]^ According to the global burden of ESKD continues to upsurge, the annual growth rate of PD utilization is anticipated to rise in parallel, especially in low- and middle-income countries with limited access to center-based hemodialysis and/or kidney transplantation.^[2]^

Despite the improvement in the practice and pharmacological treatments, cardiovascular disease still remains the most adverse outcomes, which resulted in significant morbidity and mortality, and healthcare costs in PD patients worldwide.^[3–6]^ From patient/caregiver and healthcare professional's perspectives, cardiovascular disease is the top of core outcome set for practice treatment and trials in PD population.^[7]^ With regard to traditional cardiovascular risk factors, there is increasing epidemiological evidence on the relationship between the residual kidney function (RKF) and long-term outcomes in PD patients. Existing clinical studies have revealed that RKF in terms of estimated glomerular filtration rate and urine volume declines over time relating to cardiovascular outcomes, all-mortality, and health-related quality of life in PD patients.^[8–12]^

Over the past decades, several controlled trials have illustrated that inhibition of the renin-angiotensin system can reduce cardiovascular events, cardiovascular mortality, and all-cause mortality in the general population as well as high-risk populations with mild to moderate chronic kidney disease.^[13–16]^ Currently, increasing clinical studies among PD patients confirmed that blockade of the renin-angiotensin system such an angiotensin-converting enzyme inhibitors (ACEI) and angiotensin receptor blockers (ARB) are likely to preserve RKF in these populations.^[17–22]^ In addition to the protective effect on RKF, previous controlled trials have demonstrated that the use of ACEI/ARB had beneficial effects for the suppression of pathological cardiovascular remodeling with decrease in blood pressure variability and left ventricular mass index.^[23,24]^ Based on the recommendations by the International Society for PD,^[25]^ inhibitions of renin-angiotensin system with ACEI/ARB in PD patients with significant RKF may improve patients’ survival and allow patients to be sustained on long-term PD use. However, the long-term effectiveness of renin-angiotensin system inhibitors with ACEI/ARB in PD patients has not been fully elucidated. Existing systematic reviews in PD patients have revealed that ACEI/ARB significantly benefit in preserving RKF, whereas limited evidence exists regarding the relative efficacy in terms of mortality, cardiovascular morbidity and mortality, and adverse events.^[26–29]^

To address this knowledge gap, we will conduct a systematic review and meta-analysis of randomized controlled trials (RCTs) and non-randomized studies (quasi-RCT and comparative effectiveness observational studies [cohort studies and case-control studies]) in PD patients to summarize the effectiveness of the use of ACEI/ARB on long-term all-cause mortality, cardiovascular morbidity and mortality, and adverse events. We also plan to incorporate our retrospective cohort study in Thai PD population into this systematic review to deliver more comprehensive evidence.

## 2 Methods

Our systematic review and meta-analysis will be conducted in accordance with the Cochrane collaboration handbook for systematic reviews of interventions^[30]^ and the method guide for effectiveness and comparative effective reviews, 2014 edition by the agency for healthcare research and quality.^[31]^ The pre-specified protocol has been registered in the International Prospective Register of Systematic Reviews (PROSPERO: registration number, CRD42019129492). The present protocol is reported in line with the preferred reporting items for systematic review and meta-analysis protocols (PRISMA-P) statement.^[32]^

### 2.1 Data sources and search strategy

**Figure 1:**
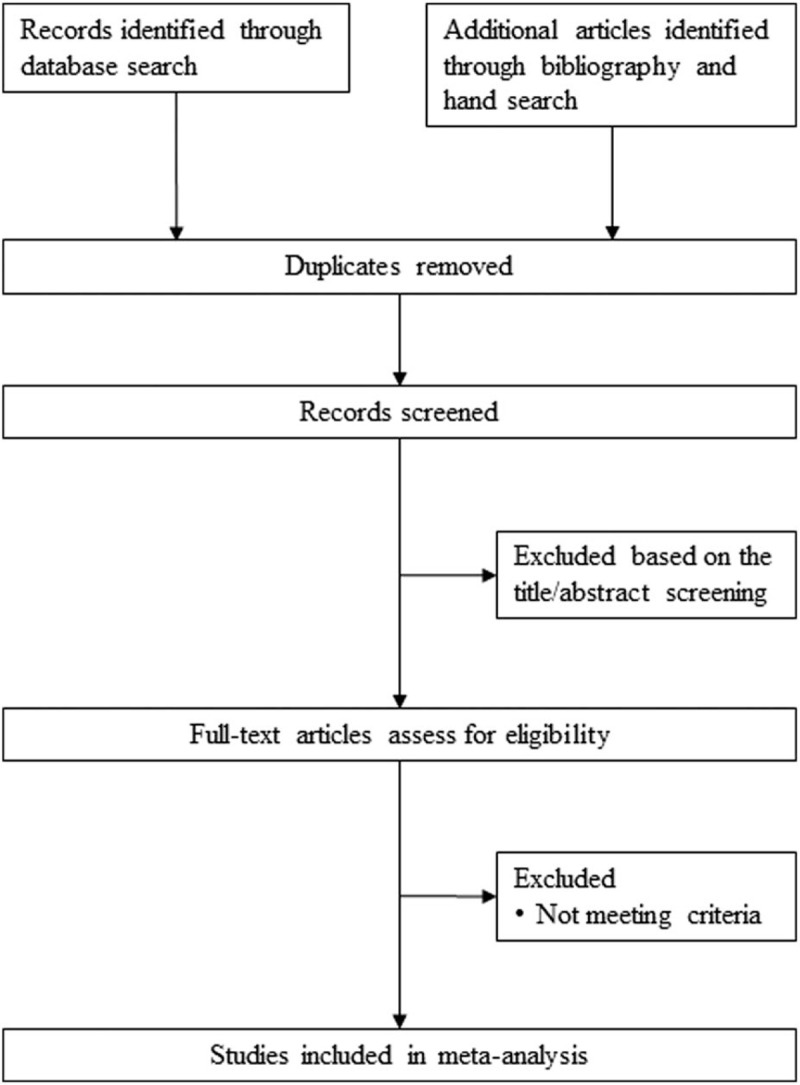
Study flow of the literature review process.

An experienced information specialist will develop electronic search strategies using an iterative process and in collaboration with the research team. Electronic databases, including PubMed, Medline, EMBASE, Cochrane Library, Web of Science, Scopus, and CINAHL will be searched from inception to February 29, 2020, with no language restrictions (Fig. 1). The search strategy will be comprised of subject headings/Medical Subject Headings terms including pharmacological class and individual ACEI/ARB (e.g., *renin-angiotensin system*, *ACEI, angiotensin II receptor blockers, benazepril, captopril, cilazapril, delapril, enalapril, fosinopril, imidapril, Lisinopril, moexipril, perindopril, quinapril, Ramipril, spirapril, temocapril, trandolapril, zofenopril, azilsartan, candesartan, eprosartan, fimasartan, irbesartan, losartan, olmesartan, tasosartan, telmisartan, valsartan*). Details of pre-specified search strategies for electronic databases are provided in Table 1  and Supplemental Digital Content Appendix 1.

**Table 1:**
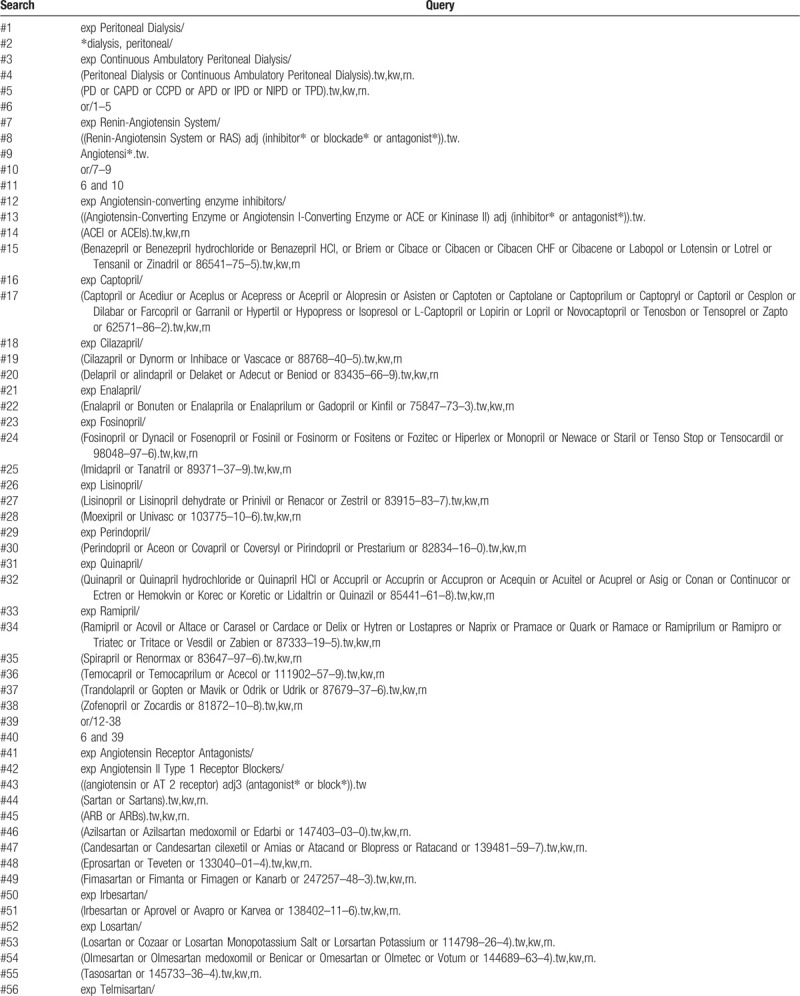
Systematic review search strategy via Ovid MEDLINE.

**Table 1 (Continued):**
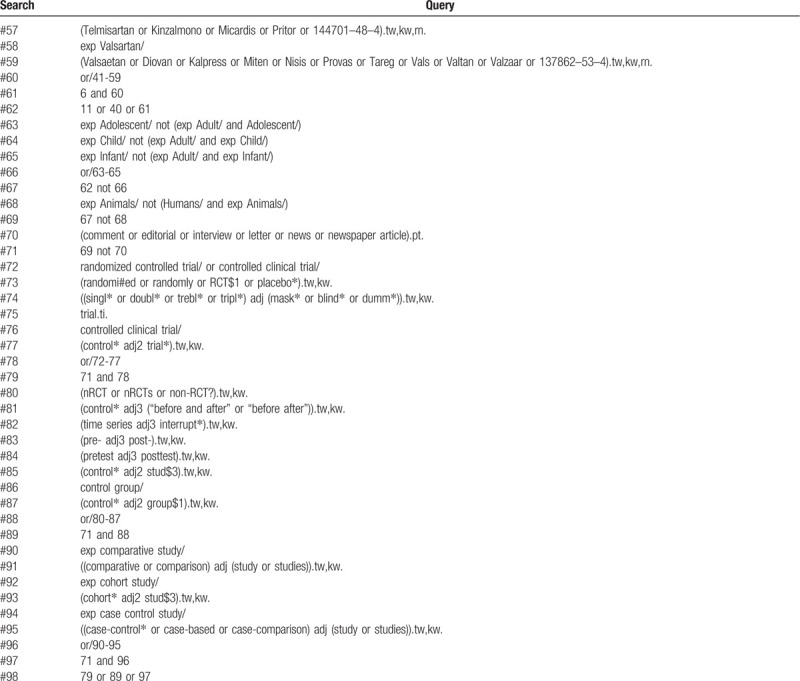
Systematic review search strategy via Ovid MEDLINE.

Grey literature from Google Scholar and clinical trial registries will be browsed for identification of additional suitable studies (Supplemental Digital Content Appendix 2). The abstracts of conference proceedings from the major international nephrology congresses (American Society of Nephrology, International Society of Nephrology, European Renal Association-European Dialysis and Transplant Association, and International Society for PD) will be searched. Moreover, reference lists of the retrieved studies, relevant guidelines, and prior systematic reviews will be manually browsed for other eligible studies.

### 2.2 Eligibility criteria and study selection process

A pair of reviewers (SN and PM) will first screen titles/abstracts identified by the literature search, and will subsequently screen potentially relevant full-text articles to establish the final set of included studies. Any discrepancy will be resolved through a team discussion and/or consultation with the third reviewer (CR). Potentially eligible studies in non-English languages will be translated before full-text appraisal. Key elements of the study design and eligibility criteria according to the PICOTS framework (population, intervention, comparison, outcome, timing, and setting) are provided in Table 2. The primary outcomes will be all-cause mortality, cardiovascular death, and cardiovascular/cerebrovascular events. Secondary outcomes of interest will be comprised of the incidence of adverse events, health status and quality of life, and healthcare utilization (Table 2).

**Table 2:**
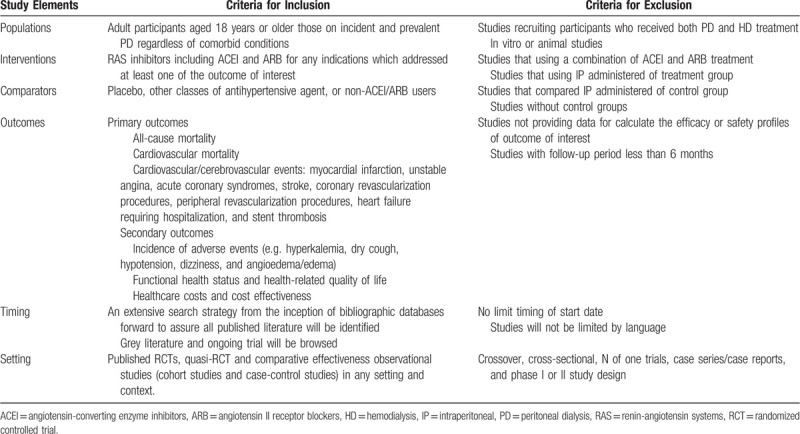
The main study elements in PICOTS format: study inclusion/exclusion criteria.

### 2.3 Data extraction

Two reviewers (SN and PM) will extract data using a standardized approach and predesigned electronic extraction form implemented in a Microsoft Excel 2016 software. The following information will be collected: study characteristics (e.g., author names, trial design, number of participants, and follow-up duration), participants’ baseline characteristics (e.g., age, sex, race, blood pressure, PD modality, history of diabetes, history of coronary artery disease), intervention and control group (individual ACEI/ARB, treatment characteristics and dosage, specific control, and concomitant medications), and predefined outcomes of interest (e.g., methods/definitions of assessment outcomes). If quantitative data are reported in figures only, the program GetData Graph Digitizer (http://getdata-graph-digitizer.com) will be used to extract numerical values from published figures.

For reasons of clinical practicality, the definition of outcomes of interest will be defined according to the study investigators or data sources (electronic medical records/administrative data) of each study. Multiple associated publications will be assembled as one single study with regard to the follow-up period, and/or the most relevant information. Two investigators (CR and RA) will verify and cross-check the data. Any disagreements will be addressed through a team discussion. For studies with incomplete data or uncertain information, the corresponding author will be contacted by email for further clarification.

### 2.4 Risk of bias

Two reviewers (SN and PM) will independently review and appraise the risk of bias for each included study accordingly to the study design. RCTs and quasi-experimental studies will be assessed by the Cochrane risk-of-bias assessment tool and then summarized as the overall risk-of-bias judgment (low risk of bias, some concerns, and high risk of bias), in which focus on bias arising from the randomization process, bias due to deviations from intended interventions, bias due to missing outcomes data, bias in measurement of the outcome, and bias in selection of the reported result.^[33]^ The Newcastle-Ottawa Scale will be applied to assess the risk of bias of comparative effectiveness observational studies (cohort or case-control studies), in which the higher scores indicate the quality of study (summary score ranging from 0–9).^[34]^

### 2.5 Data synthesis

We are planning to integrate existing studies and data from our own conducted retrospective cohort study to this systematic review and meta-analysis. Only full-text studies will be considered in the primary analysis; however, sensitivity analyses will be performed by adding relevant abstracts from conference meetings. If data are available, subgroup analyses evaluating the use of ACEI/ARB, dosage, and individual ACEI/ARB will be considered to explore for the presence of dose- and duration-response effects.

The order of preference for combining data, when multiple options are available by study authors (e.g., raw data [2 × 2 tables], unadjusted effects measures, adjusted effects measures) is provided in Table 3. A qualitative synthesis (systematic review) will be performed to summarize the findings. When applicable, the hazard ratios with the greatest degree of adjustment for potential confounding factors will be considered as the common effect estimates of association across studies. The pooled effects estimate and 95% confidence intervals (CIs) will be estimated using DerSimonian-Laird random-effects models to minimize effects of between-study heterogeneity.^[35]^ The number needed to treat will be estimated with its 95% CIs by using event rates control from our cohort as described above. Included studies with zero events will be handled by a 0.5 cell correction for binary outcomes.^[36]^ Heterogeneity will be assessed by using the Cochran *Q* test, with *P* < .10. The degree of inconsistency will be investigated by the *I*^2^ index and tau-squared (*τ*^2^) statistics, in which the heterogeneity will be estimated as low (*I*^2^ ≤ 25%, *τ*^2^ ≤ 0.01), moderate (*I*^2^ > 25% and < 75%, *τ*^2^ > 0.01 and < 0.16), and high (*I*^2^ ≥75%, *τ*^2^ ≥ 0.16).^[37]^ Visual inspection of funnel plots will be performed to investigate any evidence of publication bias. We will also assess the funnel asymmetry by using the Begg and Egger regression test, with *P* < .10.^[38,39]^ Moreover, the trim and fill method will be performed to calibrate for publication bias.^[40]^

**Table 3:**
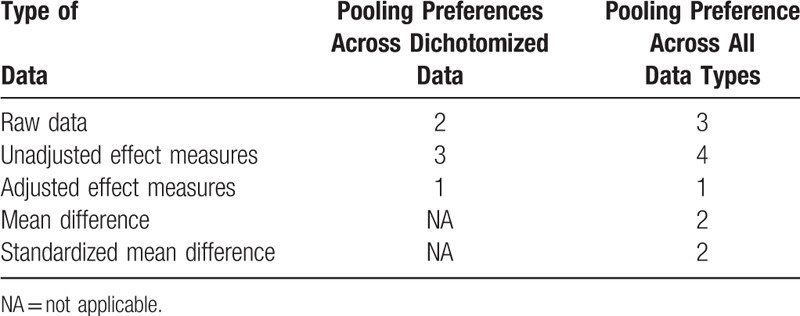
Order preference for combining data types.

Preplanned subgroup analyses and random-effects univariate meta-regressions will be performed to quantify the potential sources of heterogeneity based on studies- and patient-characteristics (e.g., study size, geographical region, age, sex, baselines blood pressure, comorbid condition [diabetes, coronary artery disease, heart failure, stroke], and PD modality). To maintain consistency of interpreting results, multiple sensitivity analyses will be considered as follows:

(i) removing individual studies approach;
(ii) stratifying the analysis according to level risk of bias, analytical methods, and study design;
(iii) adjusting for key confounding factors (age, serum albumin, blood pressure, diabetes, and history of coronary artery disease); and
(iv) post-hoc analysis by adding unpublished conference abstracts.

Statistical significance for all tests will be two-tailed, with *P* value < .05. All analyses will be performed using STATA software version 14.0 (StataCorp, College Station, TX).

### 2.6 Grading the strength of evidence

To interpret findings, 2 reviewers (SN and PM) will assess the strength of evidence for each outcome using the grading of recommended assessment, development and evaluation (GRADE) system.^[41]^ The strength of evidence will be classified as insufficient-, low-, moderate-, or high-quality evidence. Disagreements in the assessment of the risk of bias and grading of body of evidence will be resolved by discussion or by consultation of a third reviewer if necessary (CR).

## 3 Ethics and dissemination

Owing to systematic review and meta-analysis study is based on the existing published data, an ethical approval is not required. The investigators commit to report data as endorsed by the preferred reporting items for systematic reviews and meta-analyses statement guidelines^[42]^ and in line with the reporting of meta-analysis of observational studies in epidemiology guidelines^[43]^ for reporting systematic review and meta-analyses. The findings will be presented through the scientific conferences and published in peer-reviewed journals. Any modification will be succinctly described in the final report.

## 4 Discussion

Although there has been a substantial improvement in cardiovascular interventions and PD practice care in recent decades, cardiovascular disease is a leading cause of morbidity, accounting for 40% to 55% of all-cause mortality in dialysis patients in national and regional registries.^[3–6]^ Besides PD-specific factors, patients on PD treatment are at a heightened risk of developing accelerated atherosclerosis, vascular and valvular calcification, and left ventricular hypertrophy secondary to a multitude of traditional cardiovascular risk factors.^[44]^ Interestingly, evidence suggests that loss of RKF is related to all-cause mortality and may be central to the development of cardiovascular events in the PD population.^[8–12]^ For instance, the re-analysis of CANUSA (Canada-United States PD), a landmark multicenter prospective cohort of 601 incident PD patients, revealed that patient survival was associated with the magnitude of glomerular filtration rate and urine volume. Each 5 L/week/1.73 m^2^ increment in glomerular filtration rate and 250 mL increase in urine volume corresponded to a 12% and 36% decreased risk of death.^[8]^

To date, existing reviews demonstrate that ACEI/ARB significantly has benefit in preserving RKF in PD patients; however, the role of ACEI/ARB on long-term mortality, cardiovascular outcomes, and adverse events has not been fully elucidated.^[26–29]^ To our knowledge, this will be the first systematic review and meta-analysis to summarize the long-term effectiveness of ACEI/ARB in the PD population. Our study will comprise a rigorous and comprehensive approach without language restriction is anticipated to include all available evidence from the literature. However, as this study leverages both RCTs and non-RCTs, thereby, heterogeneity in study-specific estimates and differences in definitions of exposure and outcomes across studies may affect our results.

## 5 Conclusion

This systematic review and meta-analysis will summarize the effectiveness of ACEI/ARB on long-term mortality, cardiovascular outcomes, and adverse events among adult PD patients by integrated all available evidence. Evidence from this review can inform to promote the rational use of ACEI/ARB in PD practice care. We plan to disseminate our study findings in the forms of presentations at the national and international conferences as well as a peer-reviewed publication.

## Author contributions

**Conceptualization:** Surapon Nochaiwong, Chidchanok Ruengorn

**Data curation:** Surapon Nochaiwong, Pajaree Mongkhon, Ratanaporn Awiphan, Wilaiwan Chongruksut

**Formal analysis:** Surapon Nochaiwong, Chidchanok Ruengorn

**Investigation:** Surapon Nochaiwong, Chidchanok Ruengorn

**Methodology:** Surapon Nochaiwong, Chidchanok Ruengorn, Kednapa Thavorn, Brian Hutton

**Writing-original draft:** Surapon Nochaiwong, Chidchanok Ruengorn

**Writing-review & editing:** Surapon Nochaiwong, Kednapa Thavorn, Kajohnsak Noppakun, Surachet Vongsanim, Manish M. Sood, Greg A. Knoll

**Funding acquisition**: Surapon Nochaiwong

**Supervision:** Surapon Nochaiwong

Surapon Nochaiwong orcid: 0000-0003-1100-7171.

## Supplementary Material

Supplemental Digital Content
