# Supplementary material for: Effects of angiotensin-converting enzyme inhibitors or angiotensin receptor blockers on all-cause mortality, cardiovascular death, and cardiovascular events among peritoneal dialysis patients: A protocol for systematic review
Source: Medicine (Baltimore). 2020 Apr 24;99(17):e19767. doi: 10.1097/MD.0000000000019767 (PMC7220652; doi:10.1097/MD.0000000000019767)
Supplement: Supplemental Digital Content [file medi-99-e19767-s001.docx]

Supplementary table 1.The relationship between Pre-CCRT NLR status and clinicopathological factors

|  | NLR≤3.5 | NLR>3.5 | P value |
| --- | --- | --- | --- |
| c Stage |  |  |  |
| I | 24 | 7 | .203 |
| II | 91 | 39 |  |
| III | 20 | 3 |  |
| yp stage |  |  |  |
| 0 | 20 | 3 | .316 |
| I | 36 | 18 |  |
| II | 31 | 12 |  |
| III | 48 | 16 |  |
| LN metastasis |  |  |  |
| Negative | 53 | 24 | .082 |
| Positive | 46 | 10 |  |
| Grade |  |  |  |
| 1 | 6 | 1 | .712 |
| 2 | 16 | 4 |  |
| 3 | 97 | 39 |  |
| 4 | 16 | 5 |  |
| PNI |  |  |  |
| Negative | 125 | 43 | .303 |
| Positive | 10 | 6 |  |
| LVI |  |  |  |
| Negative | 117 | 43 | .846 |
| Positive | 18 | 6 |  |
| CRM |  |  |  |
| Negative | 130 | 46 | .477 |
| Close / Positive | 5 | 3 |  |
| TRG |  |  |  |
| 0–1 | 24 | 7 | .203 |
| 2–3 | 91 | 39 |  |
| 4 | 20 | 3 |  |
| CEA  <5  ≥ 5 | 66  34 | 58  27 | .747 |
